# Comparison of Bevacizumab and Aflibercept for Suppression of Angiogenesis in Human Retinal Microvascular Endothelial Cells

**DOI:** 10.3390/ph16070939

**Published:** 2023-06-29

**Authors:** Amirfarbod Yazdanyar, Charles L. Cai, Jacob V. Aranda, Eric Shrier, Kay D. Beharry

**Affiliations:** 1 Department of Ophthalmology, State University of New York, Downstate Health Sciences University, Brooklyn, NY 11203, USA; 2 Retina Group of New England, Waterford, CT 06385, USA; 3 Department of Pediatrics/Division of Neonatal-Perinatal Medicine, State University of New York, Downstate Health Sciences University, Brooklyn, NY 11203, USA

**Keywords:** angiogenesis, antioxidants, human retinal microvascular endothelial cells, intermittent hypoxia, Notch signaling, VEGF signaling, Notch

## Abstract

Bevacizumab (Avastin) is a vascular endothelial growth factor (VEGF) inhibitor that is widely used for aggressive posterior retinopathy of prematurity (APROP). Its use is associated with multiple adverse effects. Aflibercept (Eylea) is a VEGFR-1 analogue that is approved for ocular use, but its efficacy for APROP is less studied. We tested the hypothesis that Eylea is as effective as Avastin for suppression of intermittent hypoxia (IH)-induced angiogenesis. Human retinal microvascular endothelial cells (HRECs) were treated with Avastin and low- or high-dose Eylea and exposed to normoxia, hyperoxia (50% O_2_), or neonatal IH for 24, 48, or 72 h. Cells were assessed for migration and tube formation capacities, as well as biomarkers of angiogenesis and oxidative stress. Both doses of Eylea suppressed migration and tube formation in all oxygen environments, although the effect was not as robust as Avastin. Furthermore, the lower dose of Eylea appeared to be more effective than the higher dose. Eylea induced soluble VEGFR-1 (sVEGFR-1) coincident with high IGF-I levels and decreased Notch/Jagged-1, demonstrating a functional association. Given the role of VEGFR-1 and Notch as guidance cues for vascular sprouting, these data suggest that Eylea may promote normal vascular patterning in a dose-dependent manner.

## 1. Introduction

Vascular endothelial growth factor (VEGF) is a potent endothelial cell mitogen that plays a major role in normal and pathological angiogenesis. To mediate its actions, VEGF signals to tyrosine kinase receptors, namely VEGFR-1 and VEGFR-2, which are primarily expressed in endothelial cells [1], to promote angiogenesis. VEGF also signals to two other receptors, VEGFR-3 and neuropilin (NP), which are mainly expressed in lymphatic vessels and neuronal cells, respectively [2]. While VEGF has the highest affinity for binding to VEGFR-1, VEGFR-2 is the primary receptor that mediates VEGF action during angiogenesis [3,4]. NP acts as a co-receptor for VEGFR-2 to enhance the angiogenic potency of the VEGF [5]. VEGF is highly induced by hypoxia, and its angiogenic activities are rapidly initiated in response to tissue oxygen deficiency [6]. High levels of VEGF are associated with hypoxia and a high vascular density seen in tumors and retinopathy. VEGF is a vascular permeability factor; therefore, newly formed blood vessels within the hypoxic region are inherently leaky, a feature that likely enhances tumor metastasis [7] and retinal vascular overgrowth.

Anti-VEGF drugs are used off-label for the treatment of ocular diseases such as macular degeneration and diabetic retinopathy in adults and, more recently, retinopathy of prematurity (ROP) in extremely low gestational age neonates (ELGANs). Bevacizumab (Avastin) is the first anti-VEGF to be approved by the United States Food and Drug Administration (FDA) for cancer treatment [8]. It is a recombinant humanized antibody that binds all forms of VEGF-A. While Avastin is not approved by the FDA for any ocular indication, it was the first anti-VEGF drug to be used off-label for the treatment of aggressive posterior ROP (APROP) in 2007 [9] and is now the most widely used drug for the treatment of APROP [10,11]. The use of Avastin in ELGANs is associated with numerous adverse effects, including vitreous or pre-retinal hemorrhage [12], delayed bilateral retinal detachments at 1 month post-treatment [13], and retinal detachment [14]. Safety studies demonstrated numerous acute and latent retinal adverse effects [15,16,17,18,19,20,21,22]. These adverse effects may be associated with prolonged serum concentrations of Avastin concurrent with suppressed systemic VEGF levels lasting up to 60 days post intravitreal administration [23,24,25,26,27]. A more recent report showed that intravitreal Avastin was associated with a greater risk for neurodevelopmental impairment [28], confirming its ability to leak into the systemic circulation. These reports underscore the need for an alternative therapy that is as effective but with fewer adverse outcomes. Aflibercept (Eylea), a human-soluble VEGF receptor that acts as a decoy to trap VEGF, was approved for ocular use by the FDA in 2011. Eylea consists of portions of human VEGF receptor-1 (VEGFR-1) and VEGFR-2 and also binds all the VEGF isoforms [29,30]. While Eylea is not generally used in ELGANs for ROP, two clinical trials demonstrated its potential benefits [31,32], and multi-center trials are ongoing.

ROP is a leading cause of childhood blindness [33]. New insights on the mechanism(s) of ROP demonstrate a direct correlation between the frequency of intermittent hypoxia (IH) and its severity [34,35]. Therefore, we tested the hypothesis that Eylea is as effective as Avastin for suppression of IH-induced angiogenesis in human retinal microvascular endothelial cells (HRECs). Our hypothesis was tested to compare (1) the effects of Avastin and Eylea on the tube formation capacity of primary human retinal microvascular endothelial cells (HRECs) exposed to normoxia, hyperoxia, and neonatal IH; (2) the suppressive action of Avastin and Eylea on biomarkers of angiogenesis, oxidative stress, and lipid peroxidation; and (3) the dose–response effect of Eylea on tube formation, and biomarkers of angiogenesis. The primary outcome was endothelial cell function as evidenced by tube formation capacity, and the secondary outcomes were the pharmacodynamic effects, including biomarkers of angiogenesis, oxidative stress, and lipid peroxidation.

## 2. Results

### 2.1. Effects on Tube Formation Capacity

**Figure 1:**
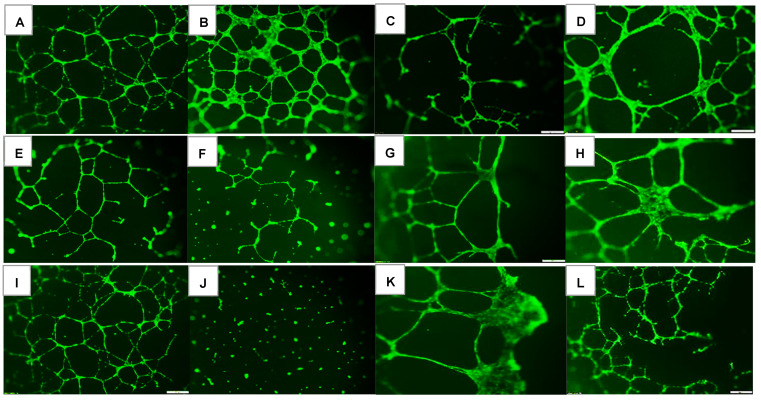
Effect of Avastin and Eylea on migration and tube formation capacities of human retinal endothelial cells exposed to normoxia (Nx, panels (**A**–**D**)), Hx (50% O_2_, panels (**E**–**H**)) or neonatal intermittent hypoxia (IH, panels (**I**–**L**)). Images were captured at 4× magnification. Scale bar is 200 µm.

The tube formation assay is widely used in in-vitro experiments to evaluate angiogenesis and the ability of vascular endothelial cells to form tubular structures. The tube formation capacity of the HRECs after 72 h is presented in Figure 1. Groups A through D are cells exposed to Nx and treated with saline (A), Avastin (B), low-dose Eylea (Lo-Eylea) (C), or high-dose Eylea (Hi-Eylea) (D). Groups E through H are cells exposed to Hx, and groups I through L are cells exposed to IH. Saline controls exposed to Nx showed normal tube formation capacities. The HRECs underwent proliferation, migration, and differentiation into networks of branching polygons and anastomosis of tubes forming a central vacuole (Figure 1A). Cells exposed to Avastin in Nx displayed thicker polygons, more branching points, and smaller vacuoles (Figure 1B). Both doses of Eylea in Nx suppressed migration and tube formation capacities of HRECs (Figure 1C,D). Exposure to Hx resulted in fewer branching points and polygons in the saline controls and Avastin-treated groups (Figure 1E,F). In contrast, Eylea treatment in Hx resulted in more polygons compared to RA but with larger vacuoles (Figure 1G,H). Neonatal IH resulted in significantly more polygons in the saline controls (Figure 1I). Treatment with Avastin suppressed the formation of tubes but not cell migration (Figure 1J), while the effects of Eylea treatment in IH on tube formation did not differ significantly from those in Hx, although significantly fewer tubes were formed compared to saline controls (Figure 1K,L). Quantitative image analysis is presented in Table 1.

### 2.2. Effect on VEGF and sVEGFR-1 Media Levels

**Figure 2:**
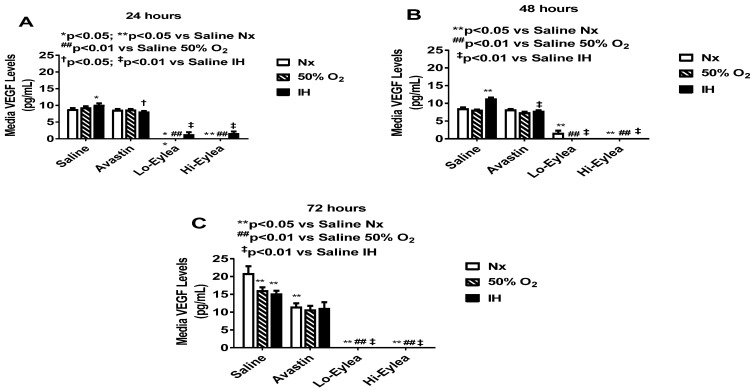
Effect of Avastin and Eylea on VEGF levels in the media of human retinal endothelial cells exposed to normoxia (Nx), Hyperoxia (Hx, 50% O_2,_), or intermittent hypoxia (IH) at 24 (**A**), 48 (**B**), and 72 (**C**) hours. Levels were normalized by cell number. Data are presented as mean ± SEM (n = 8 samples/group).

**Figure 3:**
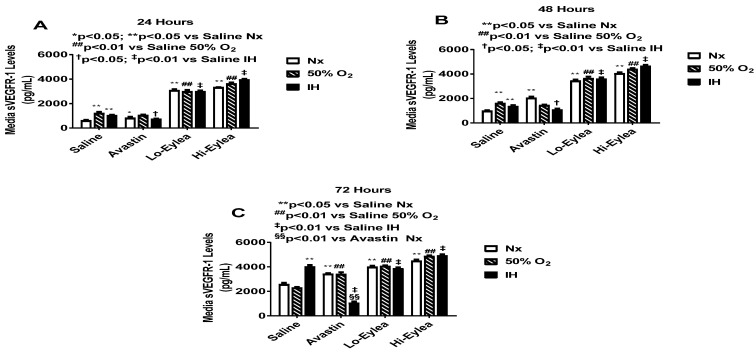
Effect of Avastin and Eylea on sVEGFR-1 levels in the media of human retinal endothelial cells exposed to normoxia (Nx), Hyperoxia (Hx, 50% O_2,_), or intermittent hypoxia (IH) at 24 (**A**), 48 (**B**), and 72 (**C**) hours. Levels were normalized by cell number. Data are presented as mean ± SEM (n = 8 samples/group).

VEGF levels in the media at 24 h, 48 h, and 72 h post-treatment are presented in Figure 2. Cells exposed to normoxia are represented by the white bar; cells exposed to hyperoxia (50% O_2_) are represented by the lined bar; and cells exposed to neonatal IH are represented by the black bar. Treatment groups are listed on the *x*-axis. At 24 h, there was no substantive difference between the media VEGF levels from the saline controls and Avastin-treated groups, except for the groups exposed to IH, which had lower media VEGF levels. In contrast, media VEGF levels were low to undetectable in the groups treated with both Eylea doses in all oxygen conditions (Figure 2A). These similar responses persisted at 48 h (Figure 2B) and 72 h (Figure 2C). At 72 h, there was an overall increase in media VEGF levels in the saline and Avastin groups. Levels of sVEGFR-1 in the media are presented in Figure 3. Soluble VEGFR-1 is a splice variant of the membrane type VEGFR-1 that acts as a VEGF trap. It binds VEGF and reduces VEGF signaling to its membrane receptor, and thereby functions as an anti-angiogenic factor. Since Eylea is a fusion protein of human VEGFR-1 and VEGFR-2, levels in the media were notably higher than Avastin, as expected, in a dose-dependent manner, in all oxygen environments. Eylea increased sVEGFR-1 in a dose-dependent manner. At 72 h, IH exposure caused a significant elevation in sVEGFR-1 levels in the saline controls and Avastin groups (Figure 3C). The media levels of sVEGFR-1 with Eylea treatment remained consistently higher at all time points. This surge in sVEGFR-1 in the saline and Avastin groups may be due to increased cell numbers over time.

### 2.3. Effect on IGF-I Media Levels

**Figure 4:**
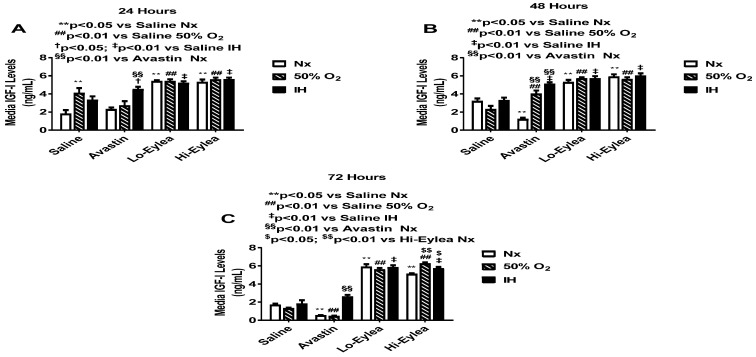
Effect of Avastin and Eylea on IGF-1 levels in the media of human retinal endothelial cells exposed to normoxia (Nx), Hyperoxia (Hx, 50% O_2,_), or intermittent hypoxia (IH) at 24 (**A**), 48 (**B**), and 72 (**C**) hours. Levels were normalized by cell number. Data are presented as mean ± SEM (n = 8 samples/group).

IGF-1 is a permissive factor for VEGF, and low levels have been shown to be associated with the development of ROP [33]. Media levels of IGF-1 at 24 h, 48 h, and 72 h are presented in Figure 4. At 24 h, media levels of IGF-I were higher in the saline controls exposed to hyperoxia and neonatal IH. Similarly, media IGF-I levels increased in the Avastin-treated group exposed to IH and in all Eylea-treated groups regardless of oxygen exposure (Figure 4A). At 48 h, there was a latent increase in IGF-I levels in the Avastin-treated group exposed to hyperoxia, while IGF-I media remained consistently higher with Avastin treatment in IH, as did groups treated with Eylea (Figure 4B). At 72 h, media IGF-I levels remained elevated only in the group treated with Avastin in IH and in all Eylea-treated groups (Figure 4C). The effect of Eylea on IGF-1 corresponded with sVEGFR-1, suggesting a functional link that may be associated with cell proliferation.

### 2.4. Effect on GSH/GSSG

**Figure 5:**
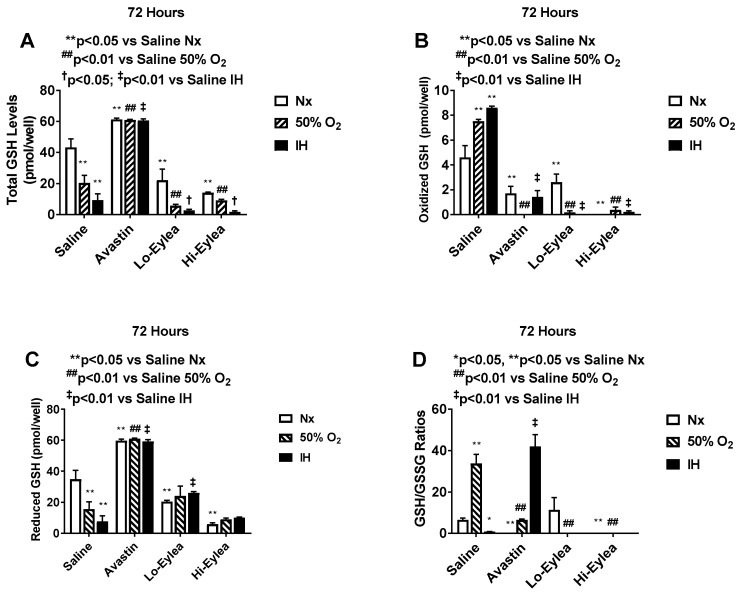
Effect of Avastin and Eylea on total GSH (**A**), oxidized GSH (**B**), reduced GSH (**C**), and GSH/GSSG ratios (**D**) in the media of human retinal endothelial cells exposed to normoxia (Nx), Hyperoxia (Hx, 50% O_2,_), or intermittent hypoxia (IH) at 72 h. Levels were normalized by cell number. Data are presented as mean ± SEM (n = 8 samples/group).

The GSH/GSSG ratio is a reliable marker for redox status [36]. Randomized control blinded studies show that reduced-oxidized glutathione ratio (GSH/GSSH) was significantly lower in asphyxiated infants receiving oxygen therapy [37]. Figure 5 shows the levels of total GSH, oxidized and reduced GSH, and GSH/GSSG ratios. GSH/GSSG ratios were elevated in the saline group exposed to hyperoxia. Avastin induced GSH/GSSG ratios in neonatal IH, while both doses of Eylea suppressed GSH/GSSG ratios in hyperoxia and neonatal IH. This was correlated with the induction of lipid peroxidation.

### 2.5. Effect on HIF_1α_

**Figure 6:**
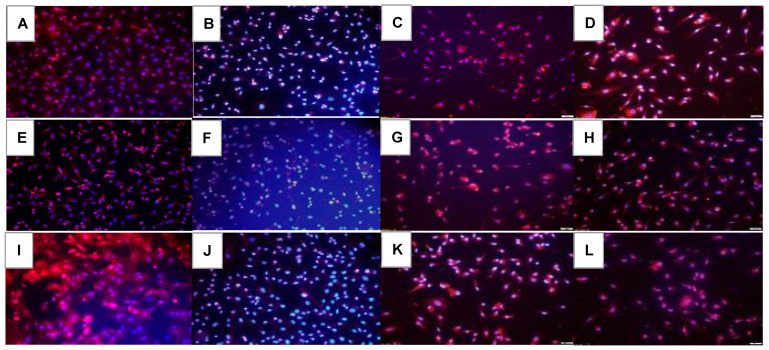
Effect of Avastin and Eylea on HIF_1α_ immunoreactivity in human retinal endothelial cells exposed to normoxia (panels (**A**–**D**)), hyperoxia (50% O_2_, panels (**E**–**H**)), or intermittent hypoxia (panels (**I**–**L**)). HIF_1α_ was determined using immunofluorescence staining (red), counterstained with DAPI (blue). Images were captured at 20× magnification. Scale bar is 100 µm.

HIF_1α_ immunoreactivity in the HRECs at 72 h is presented in Figure 6. In the saline controls, HIF_1α_ decreased in hyperoxia (Figure 6E) and increased in IH (Figure 6I). Avastin treatment caused a reduction in HIF_1α_ in all oxygen environments (Figure 6B,F,J). Exposure to Hi-Eylea in Nx caused an increased expression of HIF_1α_ in HRECs (Figure 6D) compared to Lo-Eylea (Figure 6C). Overall, an increased number of cells were noted with Avastin compared to Eylea, which appeared smaller in size. Quantitative image analysis is presented in Table 2.

### 2.6. Effect on VEGF Signaling

**Figure 7:**
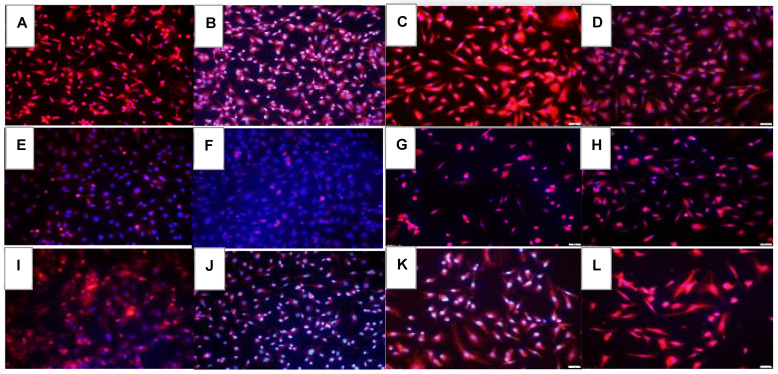
Effect of Avastin and Eylea on VEGF-A immunoreactivity in human retinal endothelial cells exposed to normoxia (panels (**A**–**D**)), hyperoxia (50% O_2_, panels (**E**–**H**)), or intermittent hypoxia (panels (**I**–**L**)). VEGF-A was determined using immunofluorescence staining (red), counterstained with DAPI (blue). Images were captured at 20× magnification. Scale bar is 100 µm.

**Figure 8:**
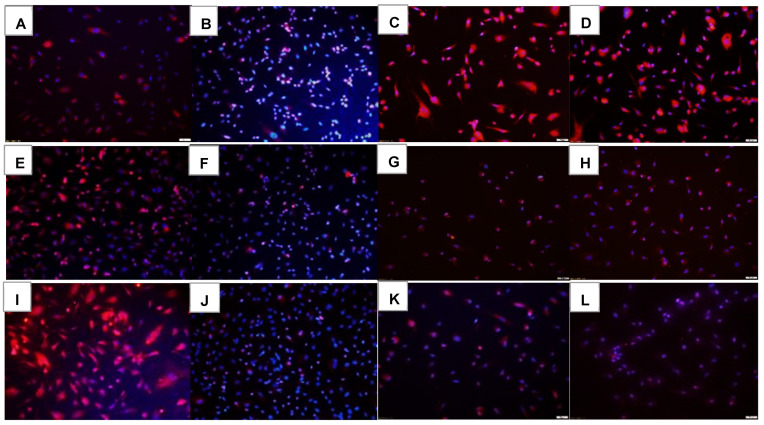
Effect of Avastin and Eylea on VEGFR-1 immunoreactivity in human retinal endothelial cells exposed to normoxia (panels (**A**–**D**)), hyperoxia (50% O_2_, panels (**E**–**H**)), or intermittent hypoxia (panels (**I**–**L**)). VEGFR-1 was determined using immunofluorescence staining (red), counterstained with DAPI (blue). Images were captured at 20× magnification. Scale bar is 100 µm.

**Figure 9:**
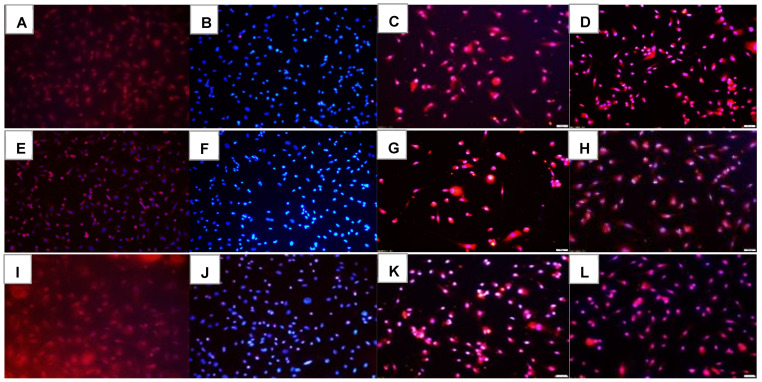
Effect of Avastin and Eylea on VEGFR-2 immunoreactivity in human retinal endothelial cells exposed to normoxia (panels (**A**–**D**)), hyperoxia (50% O_2_, panels (**E**–**H**)), or intermittent hypoxia (panels (**I**–**L**)). VEGFR-2 was determined using immunofluorescence staining (red), counterstained with DAPI (blue). Images were captured at 20× magnification. Scale bar is 100 µm.

VEGF-A immunoreactivity at 72 h is presented in Figure 7. Of the RA groups, Hi-Eylea resulted in the least expression of VEGF-A (Figure 7D). Of the groups exposed to Hx and neonatal IH, Avastin was most effective for the suppression of VEGF (Figure 7F and Figure 7J, respectively). VEGFR-1 expression is presented in Figure 8. In the saline RA controls, VEGFR-1 was not appreciably expressed (Figure 8A) and was induced with Hx (Figure 8E) and IH (Figure 8I). Compared to Eylea, Avastin suppressed VEGFR-1 expression in all oxygen conditions (Figure 8A,F,J). Both doses of Eylea decreased VEGFR-1 in Hx (Figure 8H) and IH (Figure 8L) compared to RA (Figure 8D). VEGFR-2 expression is presented in Figure 9. VEGFR-2 expression declined in the saline controls exposed to hyperoxia (Figure 9E) and increased in IH (Figure 9I). VEGFR-2 was mildly expressed with Avastin treatment (Figure 9B,F,J) but not with Eylea. Similar response patterns were noted for VEGFR-3 and Neuropilin-1 (to reduce the number of images, the data are not shown for VEGFR-3 and Neuropilin-1 due to similarities, or non-significant effects). Quantitative image analysis is presented in Table 2.

### 2.7. Effect on Notch Signaling

**Figure 10:**
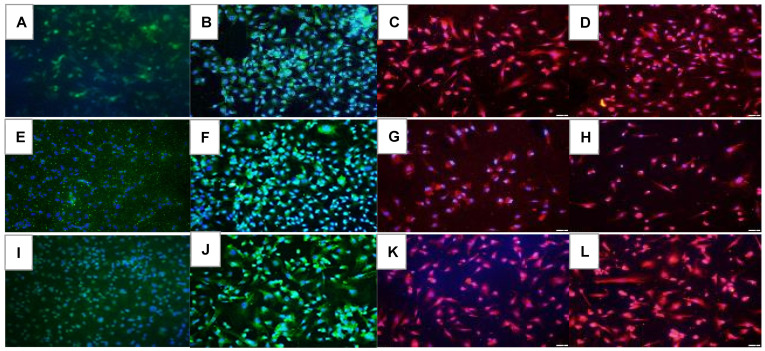
Effect of Avastin and Eylea on Notch-1 immunoreactivity in human retinal endothelial cells exposed to normoxia (panels (**A**–**D**)), hyperoxia (50% O_2_, panels (**E**–**H**)), or intermittent hypoxia (panels (**I**–**L**)). Notch-1 was determined using immunofluorescence staining. Saline and Avastin groups are stained green, and Eylea groups are stained red. Cells were counterstained with DAPI (blue). Images were captured at 20× magnification. Scale bar is 100 µm.

**Figure 11:**
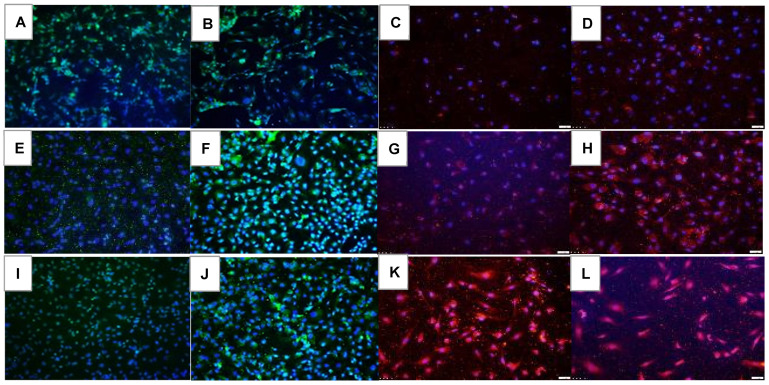
Effect of Avastin and Eylea on Notch-4 immunoreactivity in human retinal endothelial cells exposed to normoxia (panels (**A**–**D**)), hyperoxia (50% O_2_, panels (**E**–**H**)), or intermittent hypoxia (panels (**I**–**L**)). Notch-4 was determined using immunofluorescence staining. Saline and Avastin groups are stained green, and Eylea groups are stained red. Cells were counterstained with DAPI (blue). Images were captured at 20× magnification. Scale bar is 100 µm.

**Figure 12:**
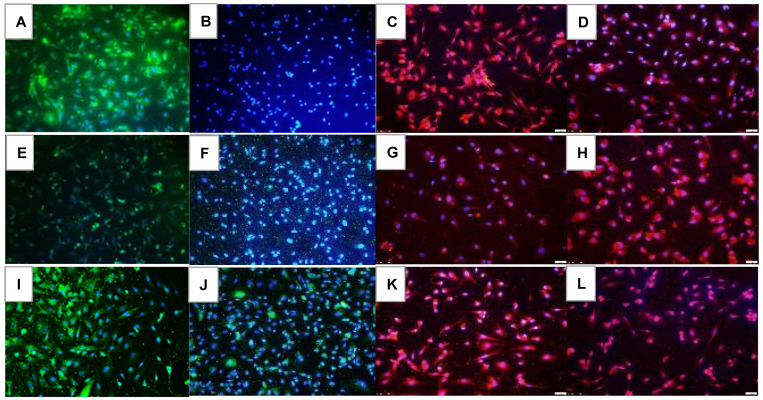
Effect of Avastin and Eylea on DLL-4 immunoreactivity in human retinal endothelial cells exposed to normoxia (panels (**A**–**D**)), hyperoxia (50% O_2_, panels (**E**–**H**)), or intermittent hypoxia (panels (**I**–**L**)). DLL-4 was determined using immunofluorescence. Saline and Avastin groups are stained green, and Eylea groups are stained red. Cells were counterstained with DAPI (blue). Images were captured at 20× magnification. Scale bar is 100 µm.

**Figure 13:**
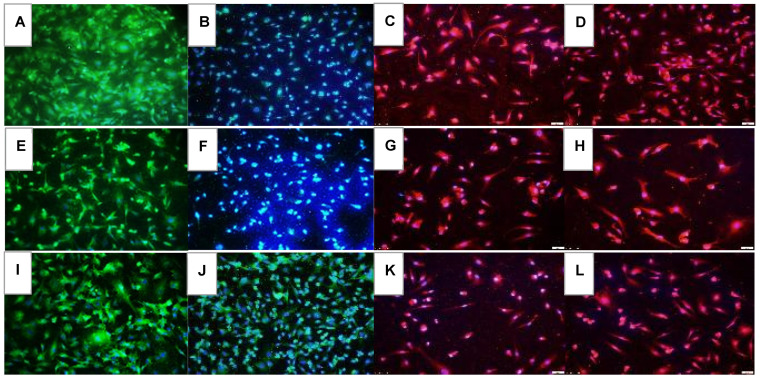
Effect of Avastin and Eylea on Jagged-1 immunoreactivity in human retinal endothelial cells exposed to normoxia (panels (**A**–**D**)), hyperoxia (50% O_2_, panels (**E**–**H**)), or intermittent hypoxia (panels (**I**–**L**)). Jagged-1 was determined using immunofluorescence. Saline and Avastin groups are stained green, and Eylea groups are stained red. Cells were counterstained with DAPI (blue). Images were captured at 20× magnification. Scale bar is 100 µm.

Notch signaling (Notch-1, Notch-2, DLL-4, and Jagged-1) at 72 h is presented in Figure 10, Figure 11, Figure 12 and Figure 13. Avastin induced Notch-1 similarly in all oxygen conditions. Both doses of Eylea similarly induced Notch-1 in the cells but to a lesser degree than Avastin (Figure 10). Notch-4 is presented in Figure 11. In RA, both doses of Eylea suppressed Notch-4 to a similar degree (Figure 11C,D). In Hx and IH, Notch-4 was decreased in the saline controls (Figure 11E and Figure 11I, respectively) and induced with Avastin (Figure 11F and Figure 11J, respectively). Notch-4 was increased with Hi-Elyea in Hx and with both doses in IH (Figure 11K,L). DLL-4 expression is presented in Figure 12. DLL-4 was highly expressed in the RA saline controls (Figure 12A). Expression levels declined with Hx exposure (Figure 12E) and resumed with IH (Figure 12I). Treatment with Avastin in RA suppressed DLL-4 (Figure 12B), but the levels increased in Hx (Figure 12F) and IH (Figure 12J). Compared to Avastin, Eylea treatment did not alter the expression of DLL-4, except for the low dose in Hx. The jagged-1 expression is presented in Figure 13. Jagged-1 was highly expressed in the RA saline controls (Figure 13A), but its expression declined in Hx (Figure 13E) and IH (Figure 13I). Avastin treatment in RA (Figure 13B) and Hx (Figure 13F) suppressed Jagged-1 compared to controls, but exposure to IH reversed the effect (Figure 13J). There were no major differences in the levels of expression of Jagged-1 among the groups treated with Eylea. Quantitative image analysis is presented in Table 2.

### 2.8. Effects on Lipid Peroxidation

**Figure 14:**
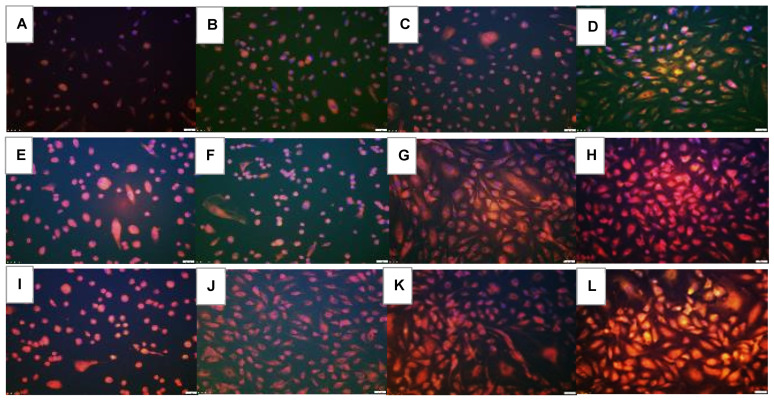
Effect of Avastin and Eylea on lipid peroxidation in human retinal endothelial cells exposed to normoxia (panels (**A**–**D**)), hyperoxia (50% O_2_, panels (**E**–**H**)), or intermittent hypoxia (panels (**I**–**L**)). Lipid peroxidation in the cells were determined using Image-iT lipid peroxidation kit. A shift from red to green indicates lipid peroxidation. Images are a combination of red, green, and blue (DAPI). Images were captured at 20× magnification. Scale bar is 100 µm.

Lipid peroxidation is the peroxidation of unsaturated lipids of cell membranes. Lipid peroxidation causes a shift from red to green and, when superimposed, appears orange. Lipid peroxidation in the cells exposed for 72 h is presented in Figure 14. In RA, the groups exposed to Avastin and Eylea, and to a greater extent, Hi-Eylea, mildly induced lipid peroxidation of HRECs at 72 h. Lipid peroxidation increased with exposure to Hx (Figure 14E,F) and IH (Figure 14I,J) in the saline controls and Avastin-treated groups, respectively. The highest levels of lipid peroxidation were noted with both doses of Eylea in Hx (Figure 14G,H), and IH (Figure 14K,L), with the highest level seen with Hi-Eylea in IH (Figure 14L, orange color). Quantitative image analysis is presented in Table 2.

## 3. Discussion

The numerous reported ocular adverse outcomes associated with the use of intravitreal Avastin in ELGANs have now extended systemically with the recent report of an increased risk for neurological disabilities [25], demonstrating that in the preterm infant, intravitreal Avastin penetrates the blood–ocular barrier and enters into the systemic circulation, with possibly devastating effects. Using the rat model exposed to neonatal IH, we showed significant retinal [38] and lung [39,40] damage with intravitreous Avastin treatment at doses similar to that being used in preterm infants. As part of our ongoing investigations, with the overarching goal of preventing severe ROP, we embarked on a series of experiments to compare and contrast the effects of Avastin with Eylea, an anti-VEGF drug that is approved for ocular use. Because ELGANs who are at the highest risk for developing severe ROP often experience frequent IH during oxygen therapy [34,35], our targets were angiogenesis, oxidative stress, and lipid peroxidation. For angiogenesis, we examined HREC functions via tube formation capacity and biomarkers that regulate and control angiogenesis. In doing so, we examined the two major pathways involved in angiogenesis, VEGF and Notch signaling pathways, to determine whether suppression of the VEGF signaling pathway results in a compensatory increase in the Notch signaling pathway, or whether the two pathways are linked and affected by anti-VEGF drugs. To establish the effects on oxidative stress and lipid peroxidation, we examined the GSH/GSSG ratios and oxidative degradation of lipids using the Image-It lipid peroxidation assay, respectively. The main findings of these novel experiments are the following: (1) Compared to saline controls, Eylea was effective for reducing the ability of HRECs to form tubes, although to a lesser degree than Avastin which did not prevent cell migration but significantly reduced tube formation capacity of the cells, particularly in IH; (2) There was a lack of dose–response effects with Eylea, with the high dose being less robust than the low dose in its ability to form tubes. However, as expected, there was a dose–response effect of increasing levels of sVEGFR-1. Given that Eylea is a VEGFR-1 analogue, higher doses induced higher sVEGFR-1 levels. (3) Eylea was most effective for decreasing the levels of VEGF in the media compared to saline and Avastin. This was most likely due to increased levels of sVEGFR-1, which acts as a VEGF trap. (4) Both doses of Eylea similarly induced IGF-I secretion by HRECs, suggesting a functional relationship, given that both sVEGFR-1 and IGF-I are produced in high amounts by ECs in culture [41,42,43,44]. Elegant studies by Lakatos D et al. [45] suggest that sVEGFR-1 is crucial for guiding blood vessel patterning. (5) Avastin was more effective than Eylea for suppressing VEGFR-2, while Eylea was more effective for suppressing Jagged-1. (6) Eylea resulted in significant lipid peroxidation and oxidative stress in the HRECs. Collectively, these data show that the low dose of Eylea is as effective as Avastin for suppression of IH-induced angiogenesis in HRECs, but the high dose, although suppressed angiogenesis compared to controls, appears to promote the normal vascular formation and patterning in a dose-dependent manner.

It is well documented that hypoxia induction of hypoxia-inducible factor (HIF)_1α_ and VEGF results in increased migration, proliferation, and invasion of retinal ECs in ocular diseases [36,37,38,39,40,41,42,43,44,45,46,47,48]. In our experiments, HRECs were not exposed to hypoxia alone. Instead, to simulate brief hypoxia experienced by ELGANs, requiring oxygen therapy for chronic lung disease, we exposed the cells to brief IH episodes with recovery in hyperoxia between each episode. Matrigel-coated wells are widely used to study the tube formation capacity of HRECs. Matrigel provides an extracellular matrix for the HRECs to migrate and form tubes and mimic angiogenesis. We noted that the saline-treated cells exposed to Hx were still capable of forming tubes, although the number of tubes was significantly reduced. An opposite effect was seen with IH exposure. Comparing the effects of Avastin and Eylea, cells exposed to Avastin produced abundant tubulogenesis, although many were pathologic and tumorigenic, appearing with thick connections and smaller central vacuoles. This effect was almost obliterated in hyperoxia. In IH, Avastin did not prevent the cells from migrating through the matrigel but completely prevented tube formation. On the other hand, while both doses of Eylea reduced tube formation capacity in all oxygen conditions compared to controls, the effect was not dose-dependent, and the high dose increased EC normal patterning, despite the reduction in tubes. VEGF signaling involves multiple VEGF ligand-receptor interactions, which include VEGF-A signaling to the VEGFR-2 and neuropilin (NP) for potent angiogenesis and vascular permeability. In our study, Avastin treatment suppressed HIF_1α_ expression in HRECs more effectively than Eylea, which had a minimal effect on HIF_1α_, although its expression was lower than the saline controls in IH. Similar suppressive effects of Avastin were seen with VEGF, VEGFR-1, and VEGFR-2. However, treatment with Eylea resulted in a significant decrease in VEGFR-1 expression. These findings provide further evidence that Avastin acts directly on the HIF/VEGF system, whereas Eylea acts via VEGFR-1, the VEGF decoy. Overall, Avastin was superior to Eylea for complete suppression of tube formation in neonatal IH, and in this context, our hypothesis of equivalent efficacy between the two drugs was not proven.

Eylea and Avastin exhibit differential VEGF binding geometry. Eylea is a VEGF “trap”. It comprises a fusion of the second and third domains of human VEGFR-1 and VEGFR-2, respectively. The main difference between Avastin and Eylea is that Eylea uniquely traps VEGF by forming a monomeric (1:1) complex that does not bind to the surface of endothelial cells. It not only blocks the amino acids necessary for VEGFR1/R2 binding but also occludes the heparin-binding site on VEGF [49]. Therefore, by trapping VEGF, Eylea makes VEGF unavailable to its receptor. In contrast, Avastin forms a large, multimeric complex that exhibits enhanced binding when complexed with heparin and NP present on the cell surface and does not block heparin binding to VEGF [49]. In our experiments, we noted very low levels of VEGF in the media, which was corrected by the number of cells. In the retina, VEGF is generally produced by astrocytes and Müller cells. However, hypoxia is known to increase the production and secretion of VEGF in many cells via the stabilization of the transcription factor HIF_1*α*_. We found that VEGF levels in the media were induced in a time-dependent manner in all saline-treated and Avastin-treated groups. This effect was completely abolished with Eylea. Concurrently, media levels of sVEGFR-1 and IGF-I remained low with saline and Avastin but increased in a dose- and time-dependent manner with Eylea. This finding was interesting, considering that IGF-I is a permissive factor for VEGF [33] and a promoter of cell proliferation [50,51]. The addition of Eylea, a fusion protein complex of VEGFR-1/VEGFR-2, to the media may have resulted in elevated sVEGFR-1, the soluble, diffused form of VEGFR-1, and, working together with IGF-I, promoted normal vascular patterning.

VEGFR-1 (also known as Flt-1) is alternatively spliced to produce both a membrane-localized form (mFlt-1) and a soluble form (sFlt-1) that is secreted by endothelial cells [42,43]. In contrast to VEGF levels, the levels of sVEGFR-1 were several times higher, confirming that HRECs predominantly secrete sVEGFR-1. Studies by Saito T et al. [52] showed that VEGF induces sVEGFR-1 in ECs by regulating the alternative splicing of the VEGFR-1 gene, indicating an interaction between VEGF and its negative regulator, sVEGFR-1. Secreted sVEGFR-1 acts as a VEGF trap but also acts as guidance cues for ECs [43], is critical for vessel morphogenesis [53,54], and VEGF-Notch crosstalk [55]. Therefore, it is reasonable to assume that the addition of Eylea to the media promoted the induction of sVEGFR-1 by the HRECs. IGF-I is a potent angiogenic factor [56] that induces VEGF mRNA in endothelial cells [57]. The concurrent induction of IGF-I and sVEGFR-1 suggests a strong positive correlation. This association has been previously reported [44,58], thus validating our findings. The exact mechanism of Eylea induction of IGF-I, coincident with sVEGFR-1, remains unknown. However, it is likely that Eylea, being a VEGFR-1 analogue, induces sVEGFR-1 in a dose-dependent manner, acting as a guidance cue for tube formation, as previously demonstrated [42,53], an effect that may be influenced by IGF-I. In this regard, it is possible, though not proven, that sVEGFR-1 and IGF-I work in tandem, as previously shown [45,58,59], to maintain and modulate EC sprouting and patterning, as demonstrated in Figure 1. This could explain the maintenance of tube formation capacity with both Eylea doses.

EC sprouting also involves a special type of cell–cell interaction [60] and involves the Notch signaling pathway and a dynamic interplay with sVEGFR-1 guidance systems [42,43,60]. There are three phenotypes of ECs, tip, stalk, and phalanx cells, which are all dependent on the binding of VEGF to its cell membrane receptors [61]. The sprouting of ECs is a highly dynamic process of switching between the tip and stalk phenotypes and involves the Notch signaling pathway [62,63,64,65]. In humans, there are four Notch receptors (Notch-1, -2, -3, and -4) and ligands (delta-like ligand, DLL-4, and Jagged-1) and five Notch ligands, including delta-like ligand (DLL)-1, -3 -4), Jagged-1, and Jagged-2. Studies show that the sprouting of EC tip cells is inhibited by Notch/DLL-4 signaling, while Notch/Jagged-1 signaling promotes potent angiogenesis and antagonizes Notch/DLL-4 [64]. Here, we show that both Avastin and Eylea induced Notch-1, while Notch-4 was induced only with Avastin in Hx and IH and with Eylea in IH. DLL-4 was highly expressed in the saline controls exposed to RA and IH but not in Hx. Although Avastin induced DLL-4 in Hx and IH, both doses of Eylea resulted in a greater DLL-4 induction in all oxygen environments. This suggests that the action of Eylea favors the Notch/DLL signaling. Induction of Notch-4/DLL-4 and suppression of Jagged-1 coincident higher sVEGFR-1 may suppress tip cell phenotype and EC sprouting. Compared to saline controls, Jagged-1 was suppressed with both Avastin and Eylea. This was reflected by the reduction in tube formation.

Another key finding was the effect of Eylea on the development of lipid peroxidation and oxidative stress in HRECs. In recent years, new information emerging from our laboratory [66] and others [67], as well as clinical studies [68,69,70], have shown that IH and oxidative stress play key roles in the development of ROP. This is due to the high susceptibility of ELGANs and their immature retinas to ROS-mediated oxidative injury caused by immature antioxidant defenses [71,72,73]. The retina is especially sensitive to oxidative stress because it is one of the highest oxygen-consuming tissues of the body, exceeding even that of the brain [74,75,76]. Our data showed that lipid peroxidation and oxidative stress (evidenced by GSH/GSSG ratios) were induced in all groups exposed to Hx and IH, but the highest induction was noted with Eylea. An association between VEGFR-1 and oxidative stress has been previously shown [77,78]. Together with those previous findings, these data suggest that Eylea suppression of EC angiogenesis may, in part, involve the induction of oxidative stress and lipid peroxidation.

## 4. Materials and Methods

### 4.1. Cells

HRECs (ACBRI-181) were purchased (Cell Systems, Kirkland, WA, USA) at 80% confluence (1.5 × 10^6^ cells) and acclimatized for 2–3 h in an incubator at 37 °C prior to plating in specialized medium of P75 flasks. Cells were activated with culture boost containing growth factors, antibiotics (Bac-Off), and 5% amphotericin B. Cell media was changed every 2 days, and the cells were passaged at 80% confluence. After 4 passages, the cells were seeded onto 24-well plates (4 × 10^4^ cells in 0.5 mL media/well) coated with an ECM product that promotes cell attachment and incubated at 37 °C, 100% humidity. The number of cells was determined with TC20 automatic cell counter (BioRad Life Sciences, Hercules, CA, USA) and were similar in each treatment and exposure group at the start of the experiment.

### 4.2. Experimental Design

Twenty-four-well plates were placed in (1) normoxia (21% O_2_; 5% CO_2_), (2) hyperoxia (Hx; 50% O_2_; 5% CO_2_), or (3) intermittent hypoxia (IH, 50% O_2_ with brief, clustered episodes of 5% O_2_; 5% CO_2_). In each oxygen environment, 3 plates (24, 48, and 72 h) were treated with either (1) bevacizumab (0.2 mg/mL), (2) low-dose Eylea (Lo-Eylea; 0.2 mg/mL), or (3) high-dose Eylea (Hi-Eylea, 0.4 mg/mL). The doses were based on a previous report of Avastin treatment in HRECs [79]. On the day of the experiment, the media was replaced with fresh media containing drug or placebo saline, and the cells were randomly assigned to the various oxygen environments. Media and cells were harvested at 24 h, 48 h, and 72 h post-treatment and frozen at −80 °C until assay. For media samples, 3 wells in each group were pooled for a total of 8 samples per group. For tube formation assays and immunofluorescence (IF) staining, cells from 6 wells per group were pooled prior to plating. Media was analyzed for VEGF, soluble VEGFR-1 (sVEGFR-1), and IGF-I levels. Cells were analyzed for tube formation capacity, lipid peroxidation, and expression of biomarkers of angiogenesis (HIF_1α_, VEGF, VEGF receptors, Notch-1, Notch-4, DLL-4, and Jagged-1).

### 4.3. Hx and Intermittent Hypoxia Profiles

Cells exposed to Hx and IH were placed into specialized dual subchambers (PROOX model 110 oxygen regulator, Biospherix, Redfield, NY, USA) attached to a C42 oxycycler (BioSpherix). The oxycycler supplied O_2_, N_2_, and CO_2_ to the subchambers according to the oxygen profile created to simulate IH [33]. The oxygen environment was monitored with oxygen sensors inside the chambers. For the Hx profile, oxygen was set continuously at 50% and remained constant until the end of the experiment. For the IH profile, oxygen was set at 50% for 30 min, followed by three 1 min hypoxia (10% O_2_) episodes, each 10 min apart, for a total of eight clustered episodes/day consistent with the severe OIR model reported by our lab [38,39,40,66]. The oxygen content in the media was continuously monitored using an oxyvalidator with an oxygen sensor (BioSpherix) inserted directly into the media of a sacrificial well with cells.

### 4.4. Tube Formation Assay

Becton Dickinson (BD)-BioCoat Angiogenesis System-EC tube formation 96-well plates (BD Biosciences, Bedford, MA, USA) were used for migration assays according to the manufacturer’s protocol. Cells from each group exposed for 72 h were harvested and plated at 2 × 10^4^ in 50 μL media in each well. Three plates were prepared, one for each oxygen environment (Nx, hyperoxia, and IH). In each 96-well plate, 24 wells were used for each treatment (saline, Avastin, Lo-Eylea, and Hi-Eylea). The plates were incubated for 16–18 h at 37 °C and 5% CO_2_ atmosphere, after which the plates were labeled with BD calcein AM fluorescent dye (BD Biosciences). The plates were imaged at 4× magnification using an Olympus BX53 microscope, DP72 digital camera, and CellSens imaging software (Olympus, Center Valley, PA, USA), attached to a Dell Precision T3500 computer (Dell, Round Rock, TX, USA). The digital images were analyzed using the count and measure tool of the CellSense imaging software. Morphometric parameters included the following: (1) total number of tubes (only fully formed tubes with complete branching polygons forming a central vacuole were counted); (2) tube length (determined as the length of each connected side of the complete polygon; (3) number of branching points (the point where the tubes three or more tubes converge; (4) thickness of the tube walls (thickness of the connecting sides of the polygons; (5) area of the central vacuole; and (6) perimeter of the central vacuole. Twenty-four images were analyzed per group to calculate mean values.

### 4.5. VEGF, sVEGFR-1 & IGF-I Assays

VEGF, sVEGFR-1, and IGF-I levels were determined in the media using commercially available human ELISA kits purchased from R & D systems (Minneapolis, MN, USA), according to the manufacturer’s protocols. Data were normalized by cell number.

### 4.6. GSH/GSSG Ratios

Levels of GSH/GSSG ratios were determined in the media using commercially available assay kits purchased from MilliporeSigma (St. Louis, MO, USA).

### 4.7. Immunofluorescence

Cells pooled from 6 wells per group were plated at the same time onto sterile 16-well culture slides (Fisher Scientific, Pittsburgh, PA, USA) and exposed to similar conditions as described above for the 24-well plates. At the end of each experimental time, 24 h, 48 h, and 72 h, the slides were washed, fixed in 4% paraformaldehyde, permeabilized, and incubated with HIF_1α_ (rabbit polyclonal IgG, 1:200, VEGF (rabbit polyclonal IgG, 1:200), VEGFR-1 (goat polyclonal IgG, 1:200), VEGFR-2 (rabbit polyclonal IgG, 1:200), VEGFR-3 (rabbit polyclonal IgG, 1:200), NP (mouse monoclonal IgG, 1:200), Notch-1 (rabbit polyclonal IgG, 1:200), Notch-4 (rabbit polyclonal IgG, 1:200), DLL-4 (rabbit polyclonal IgG, 1:200), and Jagged-1 (rabbit monoclonal IgG, 1:200 primary antibodies purchased from Invitrogen Thermo Fisher (Waltham, MA, USA), Antibodies Online (Limerick, PA, USA), MyBioSource (San Diego, CA, USA), Novus Biologicals (Centennial, CO, USA) and Santa Cruz Biotechnology (Dallas, TX, USA)). Alexa Fluor fluorescent secondary antibodies (Life Technologies, Grand Island, NY, USA). Cells were imaged at 20× magnification using an Olympus IX73 inverted microscope system and CellSens imaging software (Olympus, Center Valley, PA, USA).

### 4.8. Lipid Peroxidation

Lipid peroxidation is the oxidative degradation of cellular lipids by reactive oxygen species (ROS). Lipid peroxidation is the cause of free radical-mediated damage in cells, particularly retinal ECs. Lipid peroxidation was determined using commercially available Image-iT lipid peroxidation kit for live cells purchased from ThermoFisher Sci (Waltham, MA, USA), according to the manufacturer’s protocol. Upon oxidation in live cells, there is a shift in the fluorescence emission peak from 590 nm (red) to 510 nm (green), providing a ratiometric indication of lipid peroxidation. Cells were counterstained with DAPI (blue). Images are combination of red, green, and blue stains.

### 4.9. Statistical Analysis

To determine differences among the Nx, Hx, and IH oxygen groups and differences among the treatment groups, two-way ANOVA was used for normally distributed data and Kruskal–Wallis test for non-normally distributed data, following Bartlett’s test for normality. Post hoc analysis was performed using the Tukey or Student–Newman–Keuls test. Significance was set at *p* < 0.05, and data are reported as mean ± SEM. All analyses were two-tailed and performed using SPSS software version 16.0 (SPSS Inc., Chicago, IL, USA) and GraphPad Prism software version 5.02 (GraphPad Inc., San Diego, CA, USA).

## 5. Conclusions

Major differences between the effects of Avastin and Eylea exist predominantly due to differences in their VEGF binding dynamics, as well as the Notch signaling effects. The mechanism of action of Avastin involved direct inhibition of VEGF, while Eylea induced sVEGFR-1, which may act as a VEGF “trap”. While both doses of Eylea suppressed angiogenesis compared to the control, there was a lack of a dose–response effect in the suppression of tube formation capacity. Nevertheless, Eylea was more effective than Avastin for suppression of Notch/Jagged-1 signaling, which is known to promote angiogenesis. Collectively, although at first, high Eylea was not as robust as the low dose for suppression of tube formation, it should be noted that high Eylea also suppressed tube formation compared to controls. Due to its inherent functional nature as guidance cues for normal vessel patterning, these data suggest that Eylea may promote normal vascular patterning in a dose-dependent manner, which may involve the interaction of sVEGFR-1, IGF-I, and Notch/DLL-4. The potency and long-term safety outcomes of Avastin remain a cause for concern, particularly when used in the preterm neonate. Therefore, evidence from this study suggests that lower doses of Avastin can effectively inhibit angiogenesis while at the same time promoting normal vascular patterning. However, caution is needed since cells in culture do not exactly replicate the in vivo environment. More preclinical dose-finding studies are needed to determine the lowest Avastin dose that provides the maximum benefit with minimal or no adverse outcomes.

## Figures and Tables

**Table 1:** Morphometric Analysis of Cell Tube Formation Capacity at 72 h Post Treatment.

Groups	Number Tubes	Tube Length (μm)	Number Branching Points	Thickness of Tube Sides (μm)	Central Vacuole Diameter (μm^2^)	Central Vacuole Perimeter (μm)
***Nx:***						
Saline	50.3 ± 0.13	222.7 ± 0.33	47.0 ± 0.1	26.0 ± 0.14	40,742.8 ± 6.2	794.6 ± 0.59
Avastin	55.6 ± 0.55 **	200.3 ± 0.43 **	58.9 ± 0.3 **	45.1 ± 0.22 **	20,156.9 ± 10.3 **	556.3 ± 1.2 **
Lo-Eylea	10.0 ± 0.1 **	126.3 ± 0.3 **	29.0 ± 0.5 **	11.5 ± 0.07 **	23,213.7 ± 4.7 **	645.3 ± 0.56 **
Hi-Eylea	31.0 ± 0.2 **	181.9 ± 0.32 **	28.0 ± 0.2 **	20.5 ± 0.13 **	51,106.8 ± 8.2 **	882.9 ± 0.73 **
***50% O*_2_ *:***						
Saline	13.0 ± 0.1 ##	132.1 ± 0.29 ##	30.0 ± 0.3 ##	7.9 ± 0.05 ##	15,422.7 ± 4.6 ##	492.3 ± 0.56 ##
Avastin	12.0 ± 0.1 **##	118.3 ± 0.28 **##	37.8 ± 0.6 **##	9.2 ± 0.07 **##	25,789.8 ± 4.9 **##	641.2 ± 0.54 **##
Lo-Eylea	11.0 ± 0.2 **##	215.2 ± 0.42 **##	24.4 ± 0.1 **##	26.4 ± 0.16 **##	69,664.4 ± 9.5 **##	1073.6 ± 0.78 **##
Hi-Eylea	32.0 ± 0.15 **##	213.9 ± 0.37 **##	43.3 ± 0.6 **##	29.0 ± 0.17 **##	55,495.1 ± 7.3 **##	959.6 ± 0.66 **##
***Neonatal IH:***						
Saline	75.9 ± 0.35 ##	149.8 ± 0.29 ##	83.2 ± 0.9 ##	16.2 ± 0.12 ##	35,960.2 ± 6.2 ##	755.9 ± 0.6
Avastin	0 ± 0 **##	0 ± 0 **##	0 ± 0 **##	0 ± 0 **##	0 ± 0 **##	0 ± 0 **##
Lo-Eylea	10.0 ± 0.1 **	223.8 ± 0.43 **##	20.3 ± 0.1 **##	36.7 ± 0.25 **##	915,28.4 ± 10.1 **##	1210.1 ± 0.82 **##
Hi-Eylea	31.0 ± 0.3 **	145.2 ± 0.21 **##	62.3 ± 0.4 **##	15.9 ± 0.08 ##	32,956.5 ± 7.5 **##	699.0 ± 0.76 **##

Data are mean ± SEM (n = 24 wells per group). Nx (normoxia); IH (intermittent hypoxia); Lo-Eylea (low-dose Eylea); Hi-Eylea (high-dose Eylea). Data were analyzed using two-way ANOVA. ** *p* < 0.01 vs. Saline treatment within each oxygen environment; ## *p* < 0.01 vs. corresponding treatments in RA.

**Table 2:** Quantitative Analysis of Immunofluorescence Stains at 72 h Post Treatment.

Groups	HIF_1α_	VEGF-A	VEGFR-1	VEGFR-2	Notch-1	Notch-4	DLL-4	Jagged-1	Lipid Peroxidation
***Nx:***									
Saline	276.3 ± 0.5	591.8 ± 0.7	160.7 ± 0.5	371.0 ± 0.2	184.0 ± 0.5	211.7 ± 0.5	419.0 ± 0.3	657.7 ± 0.7	126.6 ± 0.2
Avastin	243.0 ± 0.4 **	449.5 ± 0.5 **	145.0 ± 0.7 **	106.0 ± 0.4 **	400.0 ± 0.2 **	253.3 ± 0.3 **	104.5 ± 0.3 **	270.0 ± 0.5 **	158.2 ± 0.3 **
Lo-Eylea	171.3 ± 0.4 **	440.7 ± 0.5 **	223.7 ± 0.5 **	267.3 ± 0.4 **	330.3 ± 0.5 **	133.0 ± 0.7 **	484.7 ± 1.0 **	495.3 ± 0.6 **	167.7 ± 0.3 **
Hi-Eylea	290.7 ± 0.1 **	369.3 ± 0.5 **	245.0 ± 0.7 **	322.0 ± 0.5 **	428.3 ± 0.7 **	122.3 ± 0.3 **	327.8 ± 0.7 **	459.0 ± 1.1 **	185.6 ± 0.2 **
***50% O*_2_ *:***									
Saline	232.3 ± 0.2	196.3 ± 0.3	392.3 ± 0.4	145.3 ± 0.1	167.7 ± 0.9	166.7 ± 0.2	252.3 ± 0.3	351.7 ± 0.6	132.6 ± 0.2
Avastin	109.3 ± 0.4 **	146.3 ± 0.3 **	144.0 ± 0.5 **	97.7 ± 0.3 **	466.0 ± 0.4 **	491.7 ± 0.4 **	202.0 ± 0.3 **	233.3 ± 0.5 **	192.5 ± 0.3 **
Lo-Eylea	184.0 ± 0.2 **	170.7 ± 0.2 **	147.3 ± 0.2 **	201.3 ± 0.4 **	166.3 ± 0.3 **	154.3 ± 0.2 **	305.0 ± 0.6 *#	376.7 ± 0.3 **	247.0 ± 0.2 **
Hi-Eylea	181.3 ± 03 **	219.7 ± 0.5 **	154.0 ± 0.3 **	197.7 ± 0.4 **	208.7 ± 0.4 **	182.0 ± 0.2 **	328.3 ± 0.4 **	352.3 ± 0.5 **	325.0 ± 0.5 **
***Neonatal IH:***									
Saline	528.0 ± 0.6	661.3 ± 0.6	676.0 ± 0.6	958.7 ± 1.0	211.3 ± 1.1	237.3 ± 0.6	563.0 ± 0.7	490.3 ± 0.4	230.0 ± 0.2
Avastin	282.0 ± 0.1 **	363.5 ± 0.3 **	165.5 ± 0.3 **	102.5 ± 0.2 **	384.5 ± 0.2 **	404.0 ± 0.4 **	528.5 ± 0.6 **	451.0 ± 0.3 **	201.5 ± 0.2 **
Lo-Eylea	251.3 ± 0.3 **	282.7 ± 0.5 **	127.3 ± 0.2 **	312.7 ± 0.5 **	365.3 ± 0.7 **	265.0 ± 0.4 **	590.7 ± 0.8 **	326.3 ± 0.5	293.0 ± 0.2 **
Hi-Eylea	164.0 ± 0.2 **	332.0 ± 0.2 **	154.7 ± 0.2 **	275.7 ± 0.3 **	319.3 ± 0.3 **	183.0 ± 0.4 **	499.3 ± 0.5 **	389.7 ± 0.3 **	400.8 ± 0.5 **

Data are mean ± SEM (n = 8 measurements per group). Nx (normoxia); IH (intermittent hypoxia); Lo-Eylea (low-dose Eylea); Hi-Eylea (high-dose Eylea). Data were analyzed using two-way ANOVA. * *p* < 0.05; ** *p* < 0.01 vs. Saline treatment within each oxygen environment. # *p* < 0.05 vs. Lo-Eylea RA. All groups differed significantly from their RA counterparts (*p* < 0.01).

## Data Availability

The data that support the findings of this study are available from the corresponding author upon reasonable request.

## References

[1] Ferrara N. (2001). Role of vascular endothelial growth factor in regulation of physiological angiogenesis. Am. J. Physiol. Cell Physiol..

[2] Ferrara N. (2000). VEGF: An update on biological and therapeutic aspects. Curr. Opin. Biotechnol..

[3] Takahashi H., Shibuya M. (2005). The vascular endothelial growth factor (VEGF)/VEGF receptor system and its role under physiological and pathological conditions. Clin. Sci..

[4] Olsson A.K., Dimberg A., Kreuger J., Claesson-Welsh L. (2006). VEGF receptor signalling—In control of vascular function. Nat. Rev..

[5] Soker S., Takashima S., Miao H.Q., Neufeld G., Klagsbrun M. (1998). Neuropilin-1 is expressed by endothelial and tumor cells as an isoform-specific receptor for vascular endothelial growth factor. Cell.

[6] Pugh C.W., Ratcliffe P.J. (2003). Regulation of angiogenesis by hypoxia: Role of the HIF system. Nat. Med..

[7] Robinson C.J., Stringer S.E. (2001). The splice variants of vascular endothelial growth factor (VEGF) and their receptors. J. Cell Sci..

[8] Tah V., Orlans H.O., Hyer J., Casswell E., Din N., Sri Shanmuganathan V., Ramskold L., Pasu S. (2015). Anti-VEGF Therapy and the Retina: An Update. J. Ophthalmol..

[9] Shah P.K., Narendran V., Tawansy K.A., Raghuram A., Narendran K. (2007). Intravitreal bevacizumab (Avastin) for post laser anterior segment ischemia in aggressive posterior retinopathy of prematurity. Indian J. Ophthalmol..

[10] Mintz-Hittner H.A., Kennedy K.A., Chuang A.Z., BEAT-ROP Cooperative Group (2011). Efficacy of intravitreal bevacizumab for stage 3+ retinopathy of prematurity. N. Engl. J. Med..

[11] Beharry K.D., Valencia G.B., Lazzaro D.R., Aranda J.V. (2016). Pharmacologic interventions for the prevention and treatment of retinopathy of prematurity. Semin. Perinatol..

[12] Hu J., Blair M.P., Shapiro M.J., Lichtenstein S.J., Galasso J.M., Kapur R. (2012). Reactivation of retinopathy of prematurity after bevacizumab injection. Arch. Ophthalmol..

[13] Henaine-Berra A., Garcia-Aguirre G., Quiroz-Mercado H., Martinez-Castellanos M.A. (2014). Retinal fluorescein angiographic changes following intravitreal anti-VEGF therapy. J. AAPOS.

[14] Ittiara S., Blair M.P., Shapiro M.J., Lichtenstein S.J. (2013). Exudative retinopathy and detachment: A late reactivation of retinopathy of prematurity after intravitreal bevacizumab. J. AAPOS.

[15] Jalali S., Balakrishnan D., Zeynalova Z., Padhi T.R., Rani P.K. (2012). Serious adverse events and visual outcomes of rescue therapy using adjunct bevacizumab to laser and surgery for retinopathy of prematurity. The Indian Twin Cities Retinopathy of Prematurity Screening database Report number 5. Arch. Dis. Child. Fetal Neonatal Ed..

[16] Karaca C., Oner A.O., Mirza E., Polat O.A., Sahiner M. (2013). Bilateral effect of unilateral bevacizumab injection in retinopathy of prematurity. JAMA Ophthalmol..

[17] Martínez-Castellanos M.A., Schwartz S., Hernández-Rojas M.L., Kon-Jara V.A., García-Aguirre G., Guerrero-Naranjo J.L., Chan R.V., Quiroz-Mercado H. (2013). Long-term effect of antiangiogenic therapy for retinopathy of prematurity up to 5 years of follow-up. Retina.

[18] Chablani J., Rani P.K., Balakrishnan D., Jalali S. (2014). Unusual Adverse Choroidal Reaction to Intravitreal Bevacizumab in Aggressive Posterior Retinopathy of Prematurity: The Indian Twin Cities ROP Screening (ITCROPS) Data Base Report Number 7. Semin. Ophthalmol..

[19] Tahija S.G., Hersetyati R., Lam G.C., Kusaka S., McMenamin P.G. (2014). Fluorescein angiographic observations of peripheral retinal vessel growth in infants after intravitreal injection of bevacizumab as sole therapy for zone I and posterior zone II retinopathy of prematurity. Br. J. Ophthalmol..

[20] Sato T., Wada K., Arahori H., Kuno N., Imoto K., Iwahashi-Shima C., Kusaka S. (2012). Serum concentrations of bevacizumab (avastin) and vascular endothelial growth factor in infants with retinopathy of prematurity. Am. J. Ophthalmol..

[21] Hong Y.R., Kim Y.H., Kim S.Y., Nam G.Y., Cheon H.J., Lee S.J. (2015). Plasma concentrations of vascular endothelial growth factor in retinopathy of prematurity after intravitreal bevacizumab injection. Retina.

[22] Wu W.C., Lien R., Liao P.J., Wang N.K., Chen Y.P., Chao A.N., Chen K.J., Chen T.L., Hwang Y.S., Lai C.C. (2015). Serum levels of vascular endothelial growth factor and related factors after intravitreous bevacizumab injection for retinopathy of prematurity. JAMA Ophthalmol..

[23] Kong L., Bhatt A.R., Demny A.B., Coats D.K., Li A., Rahman E.Z., Smith O.E., Steinkuller P.G. (2015). Pharmacokinetics of bevacizumab and its effects on serum VEGF and IGF-1 in infants with retinopathy of prematurity. Investig. Ophthalmol. Vis. Sci..

[24] Pieh C., Agostini H., Buschbeck C., Krüger M., Schulte-Mönting J., Zirrgiebel U., Drevs J., Lagrèze W.A. (2008). VEGF-A, VEGFR-1, VEGFR-2 and Tie2 levels in plasma of premature infants: Relationship to retinopathy of prematurity. Br. J. Ophthalmol..

[25] Morin J., Luu T.M., Superstein R., Ospina L.H., Lefebvre F., Simard M.N., Shah V., Shah P.S., Kelly E.N. (2016). Canadian Neonatal Network and the Canadian Neonatal Follow-Up Network Investigators. Neurodevelopmental Outcomes Following Bevacizumab Injections for Retinopathy of Prematurity. Pediatrics.

[26] (2008). Aflibercept: AVE 0005, AVE 005, AVE0005, VEGF Trap–Regeneron, VEGF Trap (R1R2), VEGF Trap-Eye. Drugs R&D.

[27] Dixon J.A., Oliver S.C., Olson J.L., Mandava N. (2009). VEGF trap-eye for the treatment of neovascular age-related macular degeneration. Expert. Opin. Investig. Drugs.

[28] Sukgen E.A., Söker G., Koçluk Y., Gülek B. (2017). Effect of Intravitreal Aflibercept on Central Retinal Arterial Blood Flow in Type 1 Retinopathy of Prematurity. Eur. J. Ophthalmol..

[29] Salman A.G., Said A.M. (2015). Structural, visual and refractive outcomes of intravitreal aflibercept injection in high-risk prethreshold type 1 retinopathy of prematurity. Ophthalmic Res..

[30] Wu W.C., Yeh P.T., Chen S.N., Yang C.M., Lai C.C., Kuo H.K. (2011). Effects and complications of bevacizumab use in patients with retinopathy of prematurity: A multicenter study in Taiwan. Ophthalmology.

[31] Jang S.Y., Choi K.S., Lee S.J. (2010). Delayed-onset retinal detachment after an intravitreal injection of ranibizumab for zone 1 plus retinopathy of prematurity. J. AAPOS.

[32] Suk K.K., Berrocal A.M., Murray T.G., Rich R., Major J.C., Hess D., Johnson R.A. (2010). Retinal detachment despite aggressive management of aggressive posterior retinopathy of prematurity. J. Pediatr. Ophthalmol. Strabismus.

[33] Chen J., Smith L.E. (2007). Retinopathy of prematurity. Angiogenesis.

[34] Di Fiore J.M., MacFarlane P.M., Martin R.J. (2019). Intermittent Hypoxemia in Preterm Infants. Clin. Perinatol..

[35] Di Fiore J.M., Bloom J.N., Orge F., Schutt A., Schluchter M., Cheruvu V.K., Walsh M., Finer N., Martin R.J. (2010). A higher incidence of intermittent hypoxemic episodes is associated with severe retinopathy of prematurity. J. Pediatr..

[36] Di Fiore J.M., Vento M. (2019). Intermittent hypoxemia and oxidative stress in preterm infants. Respir. Physiol. Neurobiol..

[37] Vento M., Asensi M., Sastre J., García-Sala F., Pallardó F.V., Viña J. (2001). Resuscitation with room air instead of 100% oxygen prevents oxidative stress in moderately asphyxiated term neonates. Pediatrics.

[38] Tan J.J., Cai C.L., Shrier E.M., McNally L., Lazzaro D.R., Aranda J.V., Beharry K.D. (2017). Ocular Adverse Effects of Intravitreal Bevacizumab Are Potentiated by Intermittent Hypoxia in a Rat Model of Oxygen-Induced Retinopathy. J. Ophthalmol..

[39] Valencia A.M., Cai C.L., Tan J., Duggan T.J., Valencia G.B., Aranda J.V., Beharry K.D. (2017). Intravitreal bevacizumab alters type IV collagenases and exacerbates arrested alveologenesis in the neonatal rat lungs. Exp. Lung Res..

[40] Duggan T.J., Cai C.L., Aranda J.V., Beharry K.D. (2021). Acute and chronic effects of intravitreal bevacizumab on lung biomarkers of angiogenesis in the rat exposed to neonatal intermittent hypoxia. Exp. Lung Res..

[41] Hornig C., Barleon B., Ahmad S., Vuorela P., Ahmed A., Weich H.A. (2000). Release and complex formation of soluble VEGFR-1 from endothelial cells and biological fluids. Lab. Investig..

[42] Kearney J.B., Kappas N.C., Ellerstrom C., DiPaola F.W., Bautch V.L. (2004). The VEGF receptor flt-1 (VEGFR-1) is a positive modulator of vascular sprout formation and branching morphogenesis. Blood.

[43] Chappell J.C., Taylor S.M., Ferrara N., Bautch V.L. (2009). Local guidance of emerging vessel sprouts requires soluble Flt-1. Dev. Cell.

[44] Olmos A., Díaz L., Avila E., Barrera D., López-Marure R., Biruete B., Larrea F., Halhali A. (2013). Associations between insulin-like growth factor I, vascular endothelial growth factor and its soluble receptor 1 in umbilical serum and endothelial cells obtained from normotensive and preeclamptic pregnancies. Growth Factors.

[45] Lakatos D., Somfai E., Méhes E., Czirók A. (2018). Soluble VEGFR1 signaling guides vascular patterns into dense branching morphologies. J. Theor. Biol..

[46] Arjamaa O., Nikinmaa M. (2006). Oxygen-dependent diseases in the retina: Role of hypoxia inducible factors. Exp. Eye Res..

[47] Wangsa-Wirawan N.D., Linsenmeier R.A. (2003). Retinal oxygen: Fundamental and clinical aspects. Arch. Ophthalmol..

[48] Cringle S.J., Yu D.Y. (2010). Oxygen supply and consumption in the retina: Implications for studies of retinopathy of prematurity. Doc. Ophthalmol..

[49] MacDonald D.A., Martin J., Muthusamy K.K., Luo J.K., Pyles E., Rafique A., Huang T., Potocky T., Liu Y., Cao J. (2016). Aflibercept exhibits VEGF binding stoichiometry distinct from bevacizumab and does not support formation of immune-like complexes. Angiogenesis.

[50] Schlenska-Lange A., Knüpfer H., Lange T.J., Kiess W., Knüpfer M. (2008). Cell proliferation and migration in glioblastoma multiforme cell lines are influenced by insulin-like growth factor I in vitro. Anticancer Res..

[51] Moriarty P., Boulton M., Dickson A., McLeod D. (1994). Production of IGF-I and IGF binding proteins by retinal cells in vitro. Br. J. Ophthalmol..

[52] Saito T., Takeda N., Amiya E., Nakao T., Abe H., Semba H., Soma K., Koyama K., Hosoya Y., Imai Y. (2013). VEGF-A induces its negative regulator, soluble form of VEGFR-1, by modulating its alternative splicing. FEBS Lett..

[53] Roberts D.M., Kearney J.B., Johnson J.H., Rosenberg M.P., Kumar R., Bautch B.L. (2004). The vascular endothelial growth factor (VEGF) receptor Flt-1 (VEGFR-1) modulates Flk-1 (VEGFR-2) signaling during blood vessel formation. Am. J. Pathol..

[54] Kappas N.C., Zeng G., Chappell J.C., Kearney J.B., Hazarika S., Kallianos K.G., Patterson C., Annex B.H., Bautch V.L. (2008). The VEGF receptor Flt-1 spatially modulates Flk-1 signaling and blood vessel branching. J. Cell Biol..

[55] Chappell J.C., Mouillesseaux K.P., Bautch V.L. (2013). Flt-1 (vascular endothelial growth factor receptor-1) is essential for the vascular endothelial growth factor-Notch feedback loop during angiogenesis. Arterioscler. Thromb. Vasc. Biol..

[56] Rabinovsky E.D., Draghia-Akli R. (2004). Insulin-like growth factor I plasmid therapy promotes in vivo angiogenesis. Mol. Ther..

[57] Miele C., Rochford J.J., Filippa N., Giorgetti-Peraldi S., Van Obberghen E. (2000). Insulin and insulin-like growth factor-I induce vascular endothelial growth factor mRNA expression via different signaling pathways. J. Biol. Chem..

[58] Nasioudis D., Minis E., Irani M., Kreines F., Witkin S.S., Spandorfer S.D. (2019). Insulin-like growth factor-1 and soluble FMS-like tyrosine kinase-1 prospectively predict cancelled IVF cycles. J. Assist. Reprod. Genet..

[59] Jakobsson L., Franco C.A., Bentley K., Collins R.T., Ponsioen B., Aspalter I.M., Rosewell I., Busse M., Thurston G., Medvinsky A. (2010). Endothelial cells dynamically compete for the tip cell position during angiogenic sprouting. Nat. Cell Biol..

[60] Siemerink M.J., Klaassen I., Van Noorden C.J., Schlingemann R.O. (2013). Endothelial tip cells in ocular angiogenesis: Potential target for anti-angiogenesis therapy. J. Histochem. Cytochem..

[61] Gerhardt H., Golding M., Fruttiger M., Ruhrberg C., Lundkvist A., Abramsson A., Jeltsch M., Mitchell C., Alitalo K., Shima D. (2003). VEGF guides angiogenic sprouting utilizing endothelial tip cell filopodia. J. Cell Biol..

[62] Potente M., Gerhardt H., Carmeliet P. (2011). Basic and therapeutic aspects of angiogenesis. Cell.

[63] Phng L.K., Gerhardt H. (2009). Angiogenesis: A team effort coordinated by notch. Dev. Cell.

[64] Benedito R., Roca C., Sörensen I., Adams S., Gossler A., Fruttiger M., Adams R.H. (2009). The notch ligands Dll4 and Jagged1 have opposing effects on angiogenesis. Cell.

[65] Hellstrom M., Phng L.K., Hofmann J.J., Wallgard E., Coultas L., Lindblom P., Alva J., Nilsson A.K., Karlsson L., Gaiano N. (2007). Dll4 signalling through Notch1 regulates formation of tip cells during angiogenesis. Nature.

[66] Beharry K.D., Cai C.L., Sharma P., Bronshtein V., Valencia G.B., Lazzaro D.R., Aranda J.V. (2013). Hydrogen Peroxide Accumulation in the Choroid During Intermittent Hypoxia Increases Risk of Severe Oxygen-Induced Retinopathy in Neonatal Rats. Investig. Ophthalmol. Vis. Sci..

[67] Penn J.S., Henry M.M., Wall P.T., Tolman B.L. (1995). The range of PaO_2_ variation determines the severity of oxygen-induced retinopathy in newborn rats. Investig. Ophthalmol. Vis. Sci..

[68] Martin R.J., Di Fiore J.M., Macfarlane P.M., Wilson C.G. (2012). Physiologic basis for intermittent hypoxic episodes in preterm infants. Adv. Exp. Med. Biol..

[69] Martin R.J., Wang K., Köroğlu O., Di Fiore J., Kc P. (2011). Intermittent hypoxic episodes in preterm infants: Do they matter?. Neonatology.

[70] Upton C.J., Milner A.D., Stokes G.M. (1991). Apnoea, bradycardia, and oxygen saturation in preterm infants. Arch. Dis. Child..

[71] Poggi C., Dani C. (2014). Antioxidant strategies and respiratory disease of the preterm newborn: An update. Oxid. Med. Cell Longev..

[72] Inayat M., Bany-Mohammed F., Valencia A., Tay C., Jacinto J., Aranda J.V., Beharry K.D. (2015). Antioxidants and Biomarkers of Oxidative Stress in Preterm Infants with Symptomatic Patent Ductus Arteriosus. Am. J. Perinatol..

[73] Davis J.M., Auten R.L. (2010). Maturation of the antioxidant system and the effects on preterm birth. Semin. Fetal Neonatal Med..

[74] Ames A., Li Y.Y., Heher E.C., Kimble C.R. (1992). Energy metabolism of rabbit retina as related to function: High cost of Na+ transport. J. Neurosci..

[75] Anderson B., Saltzman H.A. (1964). Retinal oxygen utilization measured by hyperbaric blackout. Arch. Ophthalmol..

[76] Yu D.Y., Cringle S.J. (2001). Oxygen distribution and consumption within the retina in vascularised and avascular retinas and in animal models of retinal disease. Prog. Retin. Eye Res..

[77] Kusmartsev S., Eruslanov E., Kübler H., Tseng T., Sakai Y., Su Z., Kaliberov S., Heiser A., Rosser C., Dahm P. (2008). Oxidative stress regulates expression of VEGFR1 in myeloid cells: Link to tumor-induced immune suppression in renal cell carcinoma. J. Immunol..

[78] Bridges J.P., Gilbert J.S., Colson D., Gilbert S.A., Dukes M.P., Ryan M.J., Granger J.P. (2009). Oxidative stress contributes to soluble fms-like tyrosine kinase-1 induced vascular dysfunction in pregnant rats. Am. J. Hypertens..

[79] Luthra S., Sharma A., Dong J., Neekhra A., Gramajo A.L., Seigel G.M., Kenney M.C., Kuppermann B.D. (2013). Effect of bevacizumab (Avastin (TM) on mitochondrial function of in vitro retinal pigment epithelial, neurosensory retinal and microvascular endothelial cells. Indian J. Ophthalmol..

